# Effects of substituting agro-industrial by-products for soybean meal on beef cattle feed utilization and rumen fermentation

**DOI:** 10.1038/s41598-022-26191-1

**Published:** 2022-12-14

**Authors:** Chaichana Suriyapha, Chanon Suntara, Metha Wanapat, Anusorn Cherdthong

**Affiliations:** Tropical Feed Resources Research and Development Center (TROFREC), Department of Animal Science, Faculty of Agriculture, Khon Kaen University, Khon Kaen, 40002 Thailand

**Keywords:** Biotechnology, Microbiology, Zoology

## Abstract

The purpose of the present investigation was to detect the effect of replacement of soybean meal (SBM) with citric waste fermented yeast waste (CWYW) as an alternative protein source of portentous substances in a concentrate mixture diet of beef cattle on intake, digestibility, ruminal fermentation, plasma urea-nitrogen, energy partitioning, and nitrogen balance. Four Thai-native beef bulls (170 ± 10.0 kg of initial body weight) were randomly allocated to a 4 × 4 Latin square design. The dietary treatments were four levels of CWYW replacing SBM in a concentrated diet at ratios of 0, 33, 67, and 100%. SBM was added to the concentrate diet at a dose of 150 g/kg DM. All cattle were offered ad libitum rice straw and the concentrate diet at 5 g/kg of body weight. The study was composed of four periods, each lasting for 21 days. The findings demonstrated that there was no difference in total dry matter intake, nutritional intake, or digestibility between treatments (p > 0.05). When CWYW replaced SBM at 100% after 4 h of feeding, ruminal pH, ammonia nitrogen, plasma urea nitrogen, and bacterial population were highest (p < 0.05). Volatile fatty acids and energy partitioning were not different (p > 0.05) among dietary treatments. Urinary nitrogen excretion was greatest (p < 0.05) for cattle fed CWYW to replace SBM at 100% of the concentrate. However, nitrogen absorption and retention for Thai-native cattle were similar (p > 0.05) among treatments. In conclusion, CWYW may be utilized as a substitute for SBM as a source of protein in Thai-native beef cattle without having an adverse impact on feed utilization, rumen fermentation characteristics, or blood metabolites.

## Introduction

The global food production chain and feed security of livestock remain a frequently well-known topic, particularly in the tropical areas during dry seasons when deficiencies of essential nutrients limit the utilization of feeds^1^. A lack of good quality protein sources, in particular, results in decreased livestock productivity^2^. Supplementation with concentrate diets is used to improve the production efficiency of animals^1,2^. Soybean meal (SBM), a substantial source of protein, is routinely added to feed mixer rations to increase the crude protein (CP) content of diets^3,4^. Soybean meal has a CP content of 420–500 g/kg^5,6^ and is high in rumen-degradable protein^7^. Nevertheless, because SBM is in high demand, the price has risen, causing greater overall feed costs^4,5^. Furthermore, increased soybean cultivation and intensified commercial crop production are frequently associated with negative environmental consequences and increased demand for natural resources, such as deforestation, soil depletion, biodiversity loss, and greenhouse gas emissions^8–10^. With the present problems of global environmental impact, global inflation, and the trend of rising feedstuff prices, there is pressure to find less expensive, more environmentally friendly feed sources of protein for livestock^4,11^. Currently, many researchers are interested in using agro-industrial by-products and residues as alternative animal feeds or utilizing biological processes such as enzyme additions or microbial fermentation that could help improve nutrient quality. Numerous of these alternatives and biological processes are viable choices for reducing feed costs and addressing the issue of environmental pollution that are both environmentally acceptable and maybe commercially viable^2,12,13^.

Recently, Suriyapha et al.^2^ developed an alternative feed product from citric waste obtained from agro-industrial citric acid processing residues fermented with yeast waste obtained from bio-ethanol processing (CWYW) as a lower-cost alternative protein source for ruminants. With a crude protein content of 535 g/kg and a true protein content of 335 g/kg, CWYW is a promising protein source for ruminant diets^2^. Furthermore, Suriyapha et al.^2^ discovered that when CWYW replaced SBM at 75% of SBM in a concentrate diet, there was no negative impact on in vitro gas kinetics, in vitro fermentation, or in vitro degradability. As a result, adopting CWYW as a substitute protein source may result in lower feed costs and industrial advantages due to its zero-waste nature.

However, researching CWYW in animals (in vivo trials) has not been reported. The study's hypothesis was that CWYW could be an alternative feed source to replace SBM without a negative impact on feed intake and feed utilization in beef cattle production. Therefore, the objective of this study was to determine the effect of replacing SBM levels with CWYW as an alternative protein source in the concentrate mixture on feed utilization, ruminal fermentation, and energy partitioning of Thai-native beef cattle fed low-quality roughage.

## Methods

All of the experimental animals and methodology involved in this research were approved by the Animal Ethics Committee under the Institutional Guidelines of Khon Kaen University, National Research Council of Thailand (record no. IACUC-KKU-27/64). Our study confirmed that all methods were performed in accordance with the relevant guidelines and regulations. The study was carried out in compliance with the ARRIVE guidelines. All experimental animals were animals under the supervision of the Tropical Feed Resources Research and Development Center (TROFREC), Khon Kaen University, which consented to this study, and all animal samples have been collected with permission.

### Preparation of citric waste fermented yeast waste (CWYW)

Citric acid waste, a by-product of the citric acid industry, was obtained from Sam Mor Farm Limited Partnership in Mueang district, Udon Thani province. Yeast waste, a by-product of ethanol production, was obtained from Green Innovation Public Company Limited (KGI) a subsidiary of Khon Kaen Sugarcane Industry Company Limited in Nam Phong district, Khon Kaen province, Thailand. The commercial-grade urea and molasses were purchased from the local feed mill in Khon Kaen province, Thailand.

CWYW was prepared according to Suriyapha et al.^2^. In brief, 100 mL of yeast waste was added to a flask and set as solution A. Then, 20 g of molasses and 50 g of urea were dissolved in 100 mL of distilled water to form solution B. After that, solutions A and B were mixed at a ratio of 1:1 (v/v). The solution’s pH was adjusted to 3.5–5 using formic acid (L.C. Industrial Co., Ltd, Nakhon Pathom, Thailand) and incubated to stimulate yeast population growth with continuous air-flush for 16 h using an air pump (HAILEA ACO-318 oxygen pump, Sagar aquarium, Gujarat, India) at room temperature. After 16 h of incubation, the media solution of yeast waste was mixed with citric acid waste at a ratio of 1:1 (v/w). Then the mixture was anaerobically fermented in 200-L plastic containers for 14-days, followed by sun-drying for 48 h to incur a moisture level of less than 10%. After drying, the CWYW was kept in plastic bags and fed to the cattle throughout the experimental period.

### The study area

The experiment was conducted at the Tropical Feed Resources Research and Development Center (TROFREC), Department of Animal Science, Faculty of Agriculture, Khon Kaen University, Khon Kaen Province (16°26′48.16′′N, 102◦49′58.8′′E), Thailand. The experiment lasted from April 2021 to July 2021 during the summer season, with a temperature of approximately 33–37 °C. All of the experimental animals were housed in the institute's research station, which is specifically designed to conduct research. As a result, the outside environment had no effect on rumination behavior or digestibility at the facility. Cattle were individually housed in four 3 × 5 m^2^ stalls with cement water tanks. Before starting the experiment, the cattle were of a similar age and body weight. The cattle were weighed, recorded as their initial body weight, dewormed (Ivomec F, Kos Introtech Co., Ltd., Bangkok, Thailand), and injected with vitamin AD_3_E (Phenix, Anitech Total Solution Co., Ltd., Bangkok, Thailand). The experimental cattle were trained in individual pens for at least 2 weeks prior to the start of the study, so the animals could adapt well to the facilities. Cattle were healthy throughout the study.

### Experimental animals, design, and dietary treatments

Four Thai native beef bulls, weighing an average of 170 ± 5.0 kg body weight (BW) were used. Cattle were randomly assigned to a 4 × 4 Latin square design. The dietary treatments were four levels of CWYW replacing SBM in a concentrate diet at ratios of 0, 33, 67, and 100%. SBM was added to the concentrate diet at a dose of 150 g/kg DM. The experimental concentrated diets were formulated following the nutritional requirements of Thai native beef cattle according to WTSR's^14^ recommendation. Each animal was offered the concentrate diet at 5 g DM/kg of BW twice a day at 07:30 am and 04:30 pm, followed by ad libitum rice straw feeding. In Table 1, the chemical compositions of the dietary concentrate mixture treatment, rice straw, SBM, and CWYW are displayed. Concentrate diets were formulated from local feedstuff with similar CP and gross energy (141 to 142 g/kg DM and 4.20 to 4.27 Mcal/kg DM, respectively) in each dietary treatment. CWYW had higher levels of CP (534 g/kg DM *vs* 457 g/kg DM), neutral detergent fiber (NDF) (392 g/kg DM *vs* 173 g/kg DM), and acid detergent fiber (ADF) (281 *vs* 133 g/kg DM) than SBM. The study was performed for four periods of 21 days each. The first 14 days were for fed adaptation, while the last 7 days were for sample collection using the total collection method in metabolism cages to assess digestibility, nitrogen excretion, fecal and urinary energy excretion. Feed intakes and refusals were recorded daily. BWs were recorded at the start and end of each period.

**Table 1:** Ingredients and chemical compositions of experimental dietary.

Item (g/kg dry matter)	SBM: CWYW				RS	SBM	CWYW
	100:0	67:33	33:67	0:100			
Cassava chips	573	575	576	578	–	–	–
Rice bran	90	90	90	90	–	–	–
Soybean meal	150	100	50	0	–	–	–
Palm kernel meal	140	140	140	140	–	–	–
CWYW	0	50	100	150	–	–	–
Urea	12	10	9	7	–	–	–
Mineral premix	10	10	10	10	–	–	–
Molasses, liquid	10	10	10	10	–	–	–
Pure sulfur	5	5	5	5	–	–	–
Salt	10	10	10	10	–	–	–
**Chemical composition**							
Dry matter (DM) (g/kg)	926	923	922	918	944	893	882
**g/kg DM**							
Organic matter	888	882	861	860	828	935	898
Ash	112	118	139	140	173	68	102
Crude protein	141	142	141	142	27	457	534
Neutral detergent fiber	159	167	171	174	781	180	401
Acid detergent fiber	86	91	96	102	539	133	293
Gross energy, Mcal/kg DM	4.35	4.32	4.30	4.28	3.75	4.02	3.89
Feed cost (USD/kg)	0.30	0.26	0.24	0.20	0.06	0.57	0.32

*SBM: CWYW* replacing soybean meal with citric waste fermented yeast waste ratio, *RS* rice straw, *SBM* soybean meal, *CWYW* citric waste fermented yeast waste.

### Sample collection and chemical analysis

The end of the experiment, cattle were transferred to the metabolism crates to study digestibility, nitrogen excretion, fecal and urinary energy excretion. Daily samples of concentrate diet, CWYW, rice straw, and refusal diet were collected and stored at − 20 °C until analysis. Fecal and urinary samples were collected, weighed, recorded, and pooled by animals for the last 7 days of each period, and stored at − 20 °C. Urinary samples were collected in a glass bottle containing H_2_SO_4_ at a 1:9 (v/v) ratio to prevent nitrogen loss and stored at − 20 °C. Samples were thawed and aliquots pooled for each animal at the end of the 7-day collection period, mixed thoroughly, and subsamples of feed offered, feed refused, and feces were dried in a forced-air oven at 60 °C for 72 h and ground to pass through a 1 mm screen using a Cyclotech Mill (Tecator, Hoganas, Sweden). Urinary samples collected during the 7-day collection period were mixed well and stored at -20 °C until analysis.

Chemical composition was analyzed according to the standard method of AOAC^15^ including dry matter (DM, no 967.03), ash (no 492.05), and crude protein (CP, no 984.13) content. The neutral detergent fiber (NDF) and acid detergent fiber (ADF) content of samples were analyzed according to the procedure of Van Soest et al.^16^. The urine nitrogen was determined according to AOAC^15^ (no. 995.04) to calculate total nitrogen excretion, apparent nitrogen absorption, and apparent nitrogen retention. The gross energy (GE) contents of the feed, feed refusal, fecal, and urinary samples were determined in an auto-calculating bomb calorimeter (SHIMADZU CA-4PJ, SHIMADZU Corporation, Japan). The digestible energy intake (DEI) was calculated from the GE of feed intake and feces. Metabolizable energy intake (MEI) was calculated as the difference between GE intake and energy loss in feces, urine, and methane energy. The methane energy prediction was estimated from the equation of Kaewpila and Sommart^17^.

On the last day of each period, blood samples were collected from the jugular vein at 0 h before and 4 h post-morning feeding. Eight milliliters of blood were collected from each animal and placed in tubes containing 12 mg of ethylenediamine tetraacetic acid (EDTA). Plasma was obtained by centrifugation at 500×*g* for 10 min and used to analyze plasma urea nitrogen (PUN) according to Crocker^18^. Ruminal fluid samples (100 mL) were collected by a stomach tube connected to a vacuum pump on the last day of the feeding trial at 0 h before and 4 h post-feeding. To avoid saliva contamination, the first three samples of ruminal fluid were discarded, and the pH of the ruminal fluid was immediately measured using a pH meter (HANNA Instruments HI 8424 microcomputer, Singapore). After measuring pH, fluid samples were separated into two parts: the first 45 mL of fluid samples were placed in a 60 ml plastic bottle containing 9 ml of 1 M H_2_SO_4_ and used to analyze for ammonia nitrogen (NH_3_-N) concentration and volatile fatty acids (VFA), including acetate (C2), propionate (C3), and butyrate (C4) concentration. The second 1 ml of ruminal fluid samples was placed in 9 ml of 10% formalin and used to enumerate the bacterial and protozoal populations. Ruminal fluid samples were centrifuged at 16,000×*g* for 15 min and the supernatant was used for NH_3_-N and VFA analysis. The concentration of ruminal NH_3_-N was analyzed according to the standard method of AOAC^15^ using the distillation technique. The concentration of VFA was analyzed using gas chromatography (DB-Wax column—30 m length, 0.25 mm diameter, 0.25 µm film; Agilent Technology, USA) as described by So et al.^19^. Bacterial and protozoal populations were counted on hemacytometers (Tiefe depth profondeur 0.1 mm and 0.0025 mm^2^, ISO LAB Laborgerate GmbH, China) using a microscope according to Galyean^20^.

### Calculations

All procedures for calculation and prediction equations of nitrogen balance, energy utilization, and methane energy were done according to the details following:

$${\text{Apparent N absorption}},{\text{ g}}/{\text{day }} = {\text{ Total N intake }}\left( {{\text{g}}/{\text{day}}} \right) \, {-}{\text{ Fecal N }}\left( {{\text{g}}/{\text{day}}} \right)$$

$${\text{Apparent N retention}},{\text{ g/day }} = {\text{ Total N intake }}\left( {{\text{g}}/{\text{day}}} \right) \, {-} \, \left( {{\text{Fecal N }}\left( {{\text{g}}/{\text{day}}} \right) \, + {\text{ Urine N }}\left( {{\text{g}}/{\text{day}}} \right)} \right)$$

$${\text{GEI}},{\text{ Mcal}}/{\text{day }} = {\text{ DM intake of diet }}\left( {{\text{kg}}/{\text{day}}} \right) \, \times {\text{ GE content in diet }}\left( {{\text{Mcal}}/{\text{kg DM}}} \right)$$

$${\text{DEI}},{\text{ Mcal}}/{\text{day }} = {\text{ GEI }}\left( {{\text{Mcal}}/{\text{day}}} \right) \, {-}{\text{ FE }}\left( {{\text{Mcal}}/{\text{day}}} \right)$$

$${\text{MEI}},{\text{ Mcal}}/{\text{day }} = {\text{ DEI }}{-} \, \left( {{\text{UE }} + {\text{Methane energy}}} \right)$$

$${\text{Methane energy}},{\text{ Mcal}}/{\text{day }} = \, \left( {{8}.{2 } \times {\text{ GEI}}} \right) \, \div { 1}00$$

where: GEI = gross energy intake; DEI = digestible energy intake; FE = fecal energy; MEI = metabolizable energy intake; UE = urine energy.

### Statistical analysis

The following equation was used to evaluate the data as a 4 × 4 Latin square design using SAS's Proc GLM procedure^21^:

$${\text{Y}}_{{{\text{ij}}}} = \, \mu \, + {\text{ T}}_{{\text{i}}} + {\text{ P}}_{{\text{j}}} + {\text{ A}}_{{\text{k}}} + \, \varepsilon_{{{\text{ijk}}}}$$

where: Y_ij_ = observations; µ = overall mean; T_i_ = substitution levels (0, 33, 67, 100%) P_j_ = effect of animal (j = 1,2,3,4); A_k_ = period (k = 1,2,3,4) and ε_ij_ = the residual effect. Results are presented as mean values with the standard error of the mean. Differences between treatment means were determined by the Duncan’s New Multiple Range Test^22^. Statistical significance was shown at *p* < 0.05 unless otherwise noted.

## Results

### Feed intake, nutrient intake, and digestibility

The effect of replacing CWYW with SBM in the concentrate diet on feed intake, nutrient intake, and digestibility of Thai-native beef cattle is shown in Table 2. Feed intake, nutrient intake, and digestibility were unaffected by substituting CWYW for SBM (0 to 100%) in the concentrate diet (p > 0.05). The total intake and dry matter digestibility ranged from 3.99 to 4.00 kg DM/day and 664.34 to 667.52 g/kg, respectively.

**Table 2:** Effect of replacing soybean meal with citric waste fermented yeast waste on feed intake and nutrient digestibility in Thai-native cattle.

Items	SBM: CWYW				SEM	p-value
	100:0	67:33	33:67	0:100		
**Rice straw intake**						
kg dry matter (DM)/day	3.11	3.11	3.10	3.10	0.02	0.89
% of body weight (BW)	1.85	1.85	1.84	1.84	0.01	0.66
g/kg BW^0.75^	66.06	66.06	65.86	65.86	0.51	0.84
**Concentrate intake**						
kg DM/day	0.89	0.89	0.89	0.89	0.003	0.56
% of BW	0.53	0.53	0.53	0.53	0.001	0.93
g/kg BW^0.75^	18.90	18.90	18.90	18.90	0.035	0.54
**Total intake**						
kg DM/day	4.00	4.00	3.99	3.99	0.57	0.26
% of BW	2.38	2.38	2.37	2.37	0.04	0.32
g/kg BW^0.75^	84.96	84.96	84.76	84.76	0.47	0.86
Feed cost (USD)/day	0.45^a^	0.42^b^	0.40^b^	0.36^c^	0.002	0.03
**Nutrients intake, kg/day**						
Organic matter	3.35	3.34	3.33	3.33	0.03	0.29
Crude protein	0.21	0.21	0.21	0.21	0.01	0.46
Neutral detergent fiber	2.56	2.58	2.59	2.58	0.02	0.57
Acid detergent fiber	1.75	1.75	1.76	1.76	0.01	0.53
**Nutrient digestibility, g/kg**						
Dry matter	667.52	665.85	664.85	664.34	6.98	0.70
Organic matter	706.92	706.84	706.53	705.73	1.27	0.55
Crude protein	615.72	609.41	607.64	607.73	6.22	0.25
Neutral detergent fiber	598.81	598.42	596.67	596.68	1.32	0.13
Acid detergent fiber	414.04	412.73	408.18	408.20	5.72	0.41

*SEM* standard error of the mean, *SBM:CWYW* replacing soybean meal with citric waste fermented yeast waste ratio, ^a,b,c^Value on the same row with different superscripts differ (p < 0.05).

### Ruminal pH, ammonia nitrogen, microbial count, and plasma urea nitrogen

The effect of replacing CWYW with SBM in the concentrate diet on ruminal ecology and plasma urea nitrogen (PUN) is shown in Table 3. Even though the mean ruminal pH was the same in all groups (p > 0.05), the inclusion of CWYW substitution of SBM in the concentrate diet at levels of 67% or above led to a higher ruminal pH (p < 0.05) at 4 h after feeding than the control group. Ruminal ammonia nitrogen (NH_3_-N) at 4 h post-feeding and the mean value were increased (p < 0.05) with the increasing CWYW replacement of SBM levels in the concentrate diet at 33% or above substitution levels. The greater PUN at 4 h post-feeding and the mean value were shown in the increasing CWYW replacement of SBM in the concentrate diet at 67% or above levels (p < 0.05). In addition, the bacterial population at 4 h post-feeding and the mean value were enhanced with increasing CWYW replacement of SBM in the concentrate diet at 67% or above (p < 0.05). However, the protozoal population did not differ (p > 0.05) among treatments with increasing substitution of SBM with CWYW levels in the concentrate diet.

**Table 3:** Effect of replacing soybean meal with citric waste fermented yeast waste on ruminal ecology and plasma urea nitrogen in Thai-native cattle.

Items	SBM: CWYW				SEM	p-value
	100:0	67:33	33:67	0:100		
**Ruminal pH**						
0 h-after feeding	6.95	6.94	6.93	6.93	0.02	0.37
4 h-after feeding	6.65^a^	6.65^a^	6.68^ab^	6.73^b^	0.03	0.04
Mean	6.80	6.80	6.80	6.82	0.02	0.89
**Ruminal NH**_**3**_**-N concentration, mg/dL**						
0 h-after feeding	10.33	10.28	10.29	10.32	0.08	0.82
4 h-after feeding	13.90^a^	14.40^b^	14.63^bc^	15.00^c^	0.11	< 0.01
Mean	12.12^a^	12.34^ab^	12.46^bc^	12.66^c^	0.07	< 0.01
**Plasma urea-N concentration, mg/dL**						
0 h-after feeding	6.50	7.00	6.75	7.00	0.49	0.46
4 h-after feeding	11.00^a^	11.00^a^	13.00^b^	13.20^b^	0.38	< 0.01
Mean	8.75^a^	9.00^a^	9.88^b^	10.10^b^	0.28	< 0.01
**Protozoal count, log10 cell/ml**						
0 h-after feeding	5.71	5.71	5.72	5.72	0.005	0.22
4 h-after feeding	6.79	6.79	6.79	6.80	0.004	0.07
Mean	6.25	6.25	6.26	6.26	0.004	0.08
**Bacterial count, log10 cell/ml**						
0 h-after feeding	9.60	9.59	9.60	9.60	0.008	0.46
4 h-after feeding	10.81^a^	10.82^a^	10.92^b^	10.93^b^	0.009	< 0.01
Mean	10.21^a^	10.20^a^	10.26^b^	10.27^b^	0.009	0.02

*SEM* standard error of the mean, *SBM: CWYW* replacing soybean meal with citric waste fermented yeast waste ratio.

^a,b,c^Value on the same row with different superscripts differ (p < 0.05).

### Ruminal volatile fatty acid concentrations

Total volatile fatty acid (VFA), acetic acid (C2), propionic acid (C3), butyric acid (C4), and C2 to C3 ratios did not change (p > 0.05) when CWYW was added to the concentrate diet in place of SBM (p > 0.05) (Table 4).

**Table 4:** Effect of replacing soybean meal with citric waste fermented yeast waste on ruminal volatile fatty acids in Thai-native cattle.

Items	SBM: CWYW				SEM	p-value
	100:0	67:33	33:67	0:100		
**Total volatile fatty acids, mM**						
0 h-after feeding	92.54	92.58	92.25	92.30	0.47	0.68
4 h-after feeding	109.10	109.13	108.68	108.78	0.60	0.68
Mean	100.82	100.86	100.47	100.54	0.53	0.69
**Acetic acid, mol/100 mol**						
0 h-after feeding	71.71	71.70	71.90	71.70	0.23	0.56
4 h-after feeding	68.14	68.12	68.35	68.15	0.25	0.55
Mean	69.93	69.91	70.13	69.93	0.24	0.55
**Propionic acid, mol/100 mol**						
0 h-after feeding	18.96	18.97	18.71	18.90	0.25	0.49
4 h-after feeding	22.53	22.52	22.23	22.44	0.28	0.50
Mean	20.75	20.75	20.47	20.67	0.27	0.50
**Butyric acid, mol/100 mol**						
0 h-after feeding	9.33	9.33	9.39	9.40	0.03	0.10
4 h-after feeding	9.33	9.36	9.42	9.41	0.04	0.10
Mean	9.33	9.35	9.41	9.41	0.04	0.11
**Acetic acid: propionic acid**						
0 h-after feeding	3.78	3.78	3.84	3.79	0.07	0.52
4 h-after feeding	3.02	3.02	3.07	3.04	0.05	0.54
Mean	3.40	3.40	3.46	3.42	0.06	0.57

*SEM* standard error of the mean, *SBM:CWYW* replacing soybean meal with citric waste fermented yeast waste ratio.

### Energy utilization and efficiency

The effect of CWYW replacing SBM meal in concentrate diet on energy utilization, energy loss, and energy efficiency of Thai-native beef cattle is shown in Table 5. There were no significant differences (p > 0.05) among dietary treatments in gross energy intake (GEI), digestible energy intake (DEI), and metabolizable energy intake (MEI) or energy loss in faeces, urine, and methane. The GEI, DEI, and MEI values ranged from 15.45 to 15.53, 9.35 to 9.44, and 6.90 to 6.98 Mcal/day, respectively. In addition, despite increasing levels of substituting CWYW for SBM in the concentrate diet, energy efficiency (DEI/GEI, MEI/GEI, and MEI/DEI) did not change across treatments (p > 0.05).

**Table 5:** Effect of replacing soybean meal with citric waste fermented yeast waste on energy utilization and efficiency in Thai-native cattle.

Items	SBM: CWYW				SEM	p-value
	100:0	67:33	33:67	0:100		
**Gross energy intake (GEI)**						
Mcal/day	15.53	15.52	15.47	15.45	0.09	0.71
Mcal/kg BW^0.75^	0.33	0.33	0.33	0.33	0.002	0.46
**Faecal energy**						
Mcal/day	6.09	6.09	6.10	6.10	0.20	0.23
% Gross energy intake	39.21	39.27	39.48	39.52	1.29	0.20
Mcal/kg BW^0.75^	0.13	0.13	0.13	0.13	0.004	0.46
**Urine energy**						
Mcal/day	1.19	1.17	1.18	1.18	0.03	0.70
% Gross energy intake	7.66	7.54	7.63	7.64	0.10	0.48
Mcal/kg BW^0.75^	0.03	0.02	0.03	0.03	0.001	0.11
**Methane energy prediction**						
Mcal/day	1.27	1.27	1.27	1.27	0.006	0.76
Mcal/kg BW^0.75^	0.03	0.03	0.03	0.03	0.001	0.85
**Digestible energy intake (DEI)**						
Mcal/day	9.44	9.43	9.37	9.35	0.34	0.24
Mcal/kg BW^0.75^	0.23	0.23	0.23	0.23	0.005	0.20
**Metabolizable energy intake (MEI)**						
Mcal/day	6.98	6.99	6.92	6.90	0.23	0.19
Mcal/kg BW^0.75^	0.15	0.15	0.15	0.15	0.004	0.16
**Energy efficiency**						
DEI/GEI	0.61	0.61	0.61	0.61	0.01	0.23
MEI/GEI	0.45	0.45	0.45	0.45	0.01	0.21
MEI/DEI	0.74	0.74	0.74	0.74	0.02	0.18

*SEM* standard error of the mean, *SBM: CWYW* replacing soybean meal with citric waste fermented yeast waste ratio.

### Nitrogen utilization efficiency

The effects of replacing CWYW with SBM in the concentrate diet on nitrogen intake, nitrogen excretion, nitrogen absorption, and nitrogen retention are reported in Table 6. The inclusion of CWYW in the concentrate diet as a substitute for SBM at 67% and 100% led to a higher urinary N excretion (p < 0.05). However, there was no observation of a mean difference between the other nitrogen utilization efficiency parameters (p > 0.05). The nitrogen intake, nitrogen absorption, and nitrogen retention ranged from 33.52 to 33.81, 20.21 to 20.54, and 12.19 to 12.57 g/day, respectively.

**Table 6:** Effect of replacing soybean meal with citric waste fermented yeast waste on nitrogen utilization and efficiency in Thai-native cattle.

Items	SBM: CWYW				SEM	p-value
	100:0	67:33	33:67	0:100		
**Nitrogen (N) intake**						
g/day	33.65	33.52	33.63	33.81	0.71	0.55
% BW	19.79	19.72	19.78	19.89	0.21	0.55
g/kg BW^0.75^	0.72	0.71	0.71	0.72	0.02	0.55
**N excretion, g/day**						
Fecal N excretion	13.11	13.31	13.37	13.37	0.34	0.57
Urinary N excretion	7.97^a^	7.95^a^	8.07^b^	8.17^b^	0.02	0.04
Apparent N absorption, g/day	20.54	20.21	20.26	20.44	0.26	0.33
Apparent N absorption, g/kg BW^0.75^	0.44	0.43	0.43	0.43	0.02	0.35
N absorption/N intake, %	61.96	60.29	60.24	61.36	0.61	0.35
Apparent N retention, g/day	12.57	12.26	12.19	12.27	0.46	0.23
Apparent N retention, g/kg BW^0.75^	0.27	0.26	0.26	0.26	0.02	0.24
N retention/N intake, %	37.37	36.57	36.25	36.29	0.52	0.24

*SEM* standard error of the mean, *SBM: CWYW* replacing soybean meal with citric waste fermented yeast waste ratio.

^a,b^Value on the same row with different superscripts differ (p < 0.05).

## Discussion

In line with earlier research, our data show that increasing the amount of CWYW in concentrate diets from 0 to 100% as a replacement for SBM had no effect on the intake of rice straw and total DM intake by cattle. Cherdthong et al.^5^ found that when yeast waste by-product obtained from the bio-ethanol process replaced SBM at 100% as a protein source in a concentrate diet, there was no effect on feed intake and nutrient digestibility in Thai-native beef cattle. Kumyos et al.^23^ reported that the supplementation of citric waste up to 30% in a concentrate diet did not affect the feed intake of Thai-native cattle. Similarly, Uriyapongson et al.^24^ found that adding up to 10% citric waste to a concentrate diet had no detrimental impact on feed intake or digestibility in Thai swamp buffaloes. However, the previous study^23–25^ did observe that the digestibility of animals decreased linearly when the amount of citric waste in a concentrate diet increased by over 10%. Suriyapha et al.^2^ also showed that replacing 100% of SBM with CWYW reduced in vitro dry matter digestibility (0.80%). Although this study did not find a decline in digestibility, this may be because the greatest level of SBM substitution with CWYW was 15% in the concentrate diet, which is close to the ideal 10% inclusion rate advised by Uriyapongson et al.^24^. The outcomes also show that when fermented citric waste with yeast waste product is administered to the cattle concentrate diet, it is comparable to SBM. Citric waste that has been fermented with yeast waste can improve flavor and digestion despite having low nutritional value^23^. Molasses-rich yeast waste may stimulate feed consumption and benefit animals, leading to an increase in feed consumption. Use of CWYW, which contains yeast cells and urea, may significantly speed up the digestion of citric waste. The previous studies by Suntara et al.^25^ and Uriyapongson et al.^24^ noted a decrease in digestibility (up to 8%) when 20 and 30% citric waste were included in the diet; they did not investigate a 15% inclusion rate. Therefore, this study result suggests that the maximum inclusion rate of CWYW is 15% of the diet. Our result that there was no significant difference in DM, NDF, and ADF intake or digestibility further suggests that CWYW should not affect intake parameters because NDF and ADF, in particular, have a major impact on feed intake restrictions and are responsible for dietary digestibility limitations^3,26^.

In the preparation process of the CWYW, urea was used as a source of nitrogen (N) which is an essential factor for yeast growth^27,28^, resulting in increased urea levels in the CWYW diet. The greater ruminal NH_3_-N concentration could be due to CWYW containing NPN-urea, which is rapidly degraded to ammonia in the rumen^29^. A increase in the concentration of NH_3_-N in the rumen is brought on by the high urea content of the CWYW and the quick hydrolysis of NH_3_-N by microbial enzymes^29,30^. In addition, changes in the proteolytic ruminal microorganism population and microbial disintegration of yeast cells into amino acids and NH_3_-N likely contributed to increased ruminal NH_3_-N in CWYW-fed animals^31,32^. Similarly, Suriyapha et al.^2^ found that the in vitro ruminal NH_3_-N concentration was increased when higher levels of CWYW replaced SBM. Ammonia-N is alkaline and its accumulation may be responsible for the increased ruminal pH^33^ at 4 h after feeding for the cattle fed CWYW. Greater ruminal pH at 4 h after feeding for cattle fed CWYW is consistent with the greater ruminal NH_3_-N concentration at 4 h after feeding. pH is an important factor that determines the activity of ruminal microbes^34^.

The monitoring PUN technique may be used to evaluate biological samples obtained at intervals that are well coordinated with the production cycle, feeding modifications, and seasonal feed availability. The greater PUN seen in cattle-fed CWYW in our study was likely a result of greater ruminal NH_3_-N production. Patra and Aschenbach^35^ revealed that the PUN concentration is related to the level of ruminal NH_3_-N production. Xu et al.^36^ demonstrated that the amount of NH_3_-N absorbed from the rumen is reflected in circulating PUN with incremental urea supplementation.

Total ruminal bacteria at 4 h post-feeding and the mean value were higher for those fed 33% or more of CWYW replacing SBM in a concentrate diet. The *S. cerevisiae* cell in CWYW is a rich source of peptides, amino acids, b-glucan, sugar, and vitamins, all of which may benefit the rumen bacterial population^37–39^. In addition, the main mechanisms for *S. cerevisiae* have been identified, and they include the stabilization of rumen pH, prevention of lactate accumulation, oxygen scavenging that results in more favorable conditions for the anaerobic communities, and nutritional competition with autochtonous ruminal species^38^. Chaucheyras-Durand et al.^40^ reported that yeast supplementation induced significant changes in relative abundances of a few bacterial species, especially *Fibrobacter succinogenes* in lambs. Similarly, Díaz et al.^41^ showed that adding yeast hydrolysate promoted ruminal bacterial community growth when compared to the non-supplemented group. The greater bacterial numbers for animals fed more than 33% CWYW could also be due to microbial growth aided by higher ruminal NH_3_-N levels^42^. The primary nitrogen source for ruminal microorganisms, ruminal ammonia is essential for peptide and amino acid synthesis, and it promotes the growth of ruminal cellulolytic and amylolytic bacteria^13,39^. The protozoal count did not vary when SBM was replaced with CWYW and did not show a negative effect on the total protozoal count. The reason for this is unknown; however, Chaucheyras-Durand et al.^43^ discovered that S. cerevisiae did not affect the total protozoal count, which could be due to *S. cerevisiae* competing with some protozoal strains for sugar utilization and being promoted in some protozoal strains to cause competition among protozoal groups, thus not affecting the total protozoal count^44^. Kowalik et al.^45^ reported that the supplementation of yeast metabolites increased *Entodinium*, whereas yeast supplementation did not affect *Diplodinium*, *Ophryscolex*, or the total protozoal population when compared with the control group. While Arakaki et al.^46^ reported a lower number of *Entodinium* and an increase in *Dasytrichia* when yeast was fed. Similarly, Cherdthong et al.^5^ reported that substituting SBM with yeast waste did not have a negative effect on the number of protozoa in Thai-native bulls.

The majority of total ruminal VFA production is related to feed digestibility, where increased digestibility leads to increased utilization of nutrients by microorganisms, and increased production of fermentation end-products^47,48^. The fact that there were no variations in DM and nutrient digestibility across the dietary treatments may be the cause of the absence of changes in total VFA production and their profile in the current investigation. It is most likely due to the fact that all dietary treatments were changed to have comparable nutritional contents in each group as there were no variations in DM intake. Additionally, there were no variations between the SBM and CWYW treatments' nutritional levels, which supports the findings without impacting the changes in digestibility and VFA generation. The in vitro study of Suriyapha et al.^2^ found that replacing SBM with CWYW in the concentrate diet had no influence on the soluble or insoluble fraction of gas production, indicating that the amount of soluble carbohydrate content was similar in each dietary treatment^49^. Similar soluble carbohydrate content among SBM and CWYW treatments is consistent with a lack of change in VFA production and profile. Similarly, Cherdthong et al.^5^ reported that the substitution of SBM with yeast waste showed no effect on VFA and molar portions of VFA.

This study's results indicated that replacing SBM with CWYW in Thai-native beef cattle did not show a negative impact on the energy utilization and resulted in the animals' gaining sufficient energy to maintain their body and growth. Similar ruminal volatile fatty acid concentrations, particularly propionic acid, between treatments may explain why there was no change in energy consumption. Propionic acid is the major primary substrate for ruminants in the gluconeogenesis reaction, which is converted into glucose as the primary energy source in the body^50^. WTSR^14^ reported that the optimal value of metabolizable energy for maintenance (MEm) in Thai-native cattle was 0.12 Mcal/kg BW^0.75^/d. In this study, the MEI of experimental animals was 0.15 Mcal/kg BW^0.75^/day, which is in the range of 0.14 to 0.19 Mcal/kg BW^0.75^/day investigated by Tangjitwattanachai and Sommart^51^ for Thai-native beef cattle. Our results suggest that energy utilization and efficiency were similar among treatments. The identical DM intake and digestibility, as well as the similar gross energy intake and energy loss in feces, urine, and methane emission, might all account for this. Similarly, Crossland et al.^52^ supplemented active dry yeast (*Saccharomyces cerevisiae*) to finishing steers and observed enhanced digestibility, lower energy loss in faeces, as well as improved ME intake. The energy efficiency values (DEI/GEI = 0.61; MEI/GEI = 0.45) we observed were close to the energy efficiency range of Thai native beef cattle fed with cassava industrial by-products in the diet^53^. Similarly, Cherdthong et al.^54^ demonstrated the DEI/GEI and MEI/GEI values ranging from 0.62 to 0.69 and 0.33 to 0.43 for Thai native bulls that consumed ensiled rice straw with or without additives, respectively. Therefore, substituting CWYW for SBM in the concentrate component of the diet might maintain Thai-native beef cattle utilizing their energy.

In this study, the N intake ranged from 0.71 to 0.72 g of N/kg BW^0.75^/day, which was close to the optimal recommended value for maintenance protein intake (0.68 g of N/ kg BW^0.75^/day) of Thai-native male cattle fed rice straw-based diets reported by Paengkoum^55^. Our results show that the nitrogen faecal excretion was similar among treatments. Cherdthong et al.^56^ explain that the levels of N intake and excretion are related to concentrates containing higher CP levels. Similarly, Gunun et al.^57^ demonstrated that greater N intake resulted in greater urinary N loss. The greater urinary N excretion in cattle fed 67 and 100% CWYW replaced SBM in the current study. It could be brought on by an increase in urea from CWYW. The rapid hydrolysis of ruminal NH_3_-N and escape from the rumen may be the cause of the increase in urinary N loss associated with increased urea levels in the CWYW diets^58^. However, replacing SBM with CWYW in the concentrate diet did not affect N absorption or N retention. The average values of N absorption and N retention were 20.36 g/d (20.21 to 20.54) and 12.32 g/d (12.19 to 12.57), respectively, which were close to the range for Thai-native cattle fed with rice straw-based diets^52,54,59,60^. Feed cost were significantly reduced when SBM was replaced with 100% CWYW, by 0.09 USD/day. This might be because CWYW costs less per kilogram than SBM ($0.32 *vs*. $0.57).

This study suggests that an alternative protein source for ruminants could be obtained by substituting citric waste fermented yeast waste (CWYW) for soybean meal (SBM) in a concentrate mixture diet. Intake, digestibility, ruminal fermentation characteristics, PUN, energy partitioning, and nitrogen balance of Thai-native cattle were not adversely affected by the substitution of SBM with CWYW up to 100%. As a result, CWYW might be employed as a protein source element, lowering farmers' production costs while simultaneously offering industrial advantages due to its zero-waste system. Future research on the impact of CWYW as a substitute for SBM should be done, nevertheless, to ascertain how production will respond under actual production conditions. However, there was no evaluation of greater levels of CWYW than in the present study, which remains a challenge to further study and evaluate the utilization of CWYW in animals and might affect different results from this study.

## Data Availability

The datasets generated and analyzed during the current study are available from the corresponding author on reasonable request.
